# Association of Total Dietary Intake of Sugars with Prostate-Specific Antigen (PSA) Concentrations: Evidence from the National Health and Nutrition Examination Survey (NHANES), 2003-2010

**DOI:** 10.1155/2021/4140767

**Published:** 2021-01-09

**Authors:** Zhangcheng Liu, Chi Chen, Fuxun Yu, Dongbo Yuan, Wei Wang, Ke Jiao, Shengbang Yang, Yongqiang Zhang, Yong Wang, Linhai Liu, Huali Xu, Yang Zhang, Guohua Zhu, Bin Hu, Jianguo Zhu

**Affiliations:** ^1^Department of Urology, Guizhou Provincial People's Hospital, The Affiliated Hospital of Guizhou Medical University, Guiyang, Guizhou Province 550002, China; ^2^Department of Urology, The Second People's Hospital of Neijiang, Neijiang, Sichuan Province 641000, China; ^3^Department of Immunology and Microbiology, Guiyang College of Traditional Chinese Medicine, Guiyang, Guizhou Province 550001, China; ^4^The National Health Commission's Key Laboratory of Immunological Pulmonary Disease, Guizhou Provincial People's Hospital, Guiyang, Guizhou Province 550002, China; ^5^Department of Urology, Guangdong Key Laboratory of Clinical Molecular Medicine and Diagnostics, Guangzhou First Municipal People's Hospital, Guangzhou Medical University, Guangzhou, Guangdong Province 510180, China

## Abstract

**Background:**

There is increasing evidence that dietary intake of sugars may be a risk factor for prostate cancer (PCa) and elevate the concentration of serum prostate-specific antigen (PSA). However, there is limited evidence of the correlation between total dietary intake of sugars and serum PSA concentrations for adult American males. Herein, we evaluated the association between total dietary intake of sugars and serum PSA concentrations in men without a malignant tumor diagnosis in the United States (US) National Health and Nutrition Examination Survey (NHANES) database. *Material and Methods*. In this secondary data analysis, a total of 6,403 men aged ≥40 years and without malignant tumor history were included from 2003 to 2010. The independent variable of this study was the total dietary intake of sugars, and the dependent variable was serum PSA concentrations. Covariates included dietary, comorbidity, physical examination, and demographic data.

**Results:**

The average age of participants included in this study was 58.1 years (±13.6). After adjusting for the dietary, comorbidity, physical examination, and demographic data, we observed that a dietary intake increase of one gram of total dietary intake of sugars was associated with an increase of serum PSA concentrations by 0.003 ng/mL (after log2 transformed, 95% CI: 0.001 to 0.005) with a *P* value for trend less than 0.05. Sensitivity analysis using the generalized additive model (GAM) supported the linear association between total dietary intake of sugars and serum PSA concentrations.

**Conclusion:**

The total dietary intake of sugars is independently and positively associated with serum PSA concentrations in adult American males who are without a personal history of malignant tumors.

## 1. Background

Prostate cancer (PCa) is the most commonly diagnosed tumor among men and the second most common cause of male death from cancer. As the population ages, its prevalence is increasing in developed countries [1]. Widespread use of prostate-specific antigen (PSA) concentrations assays has greatly improved the detection rate of highly differentiated, small, and asymptomatic PCa [1, 2]. The purpose of screening PCa with serum PSA concentrations is to detect PCa at an early intervenable stage, where it is more likely to be amenable to curative treatment and to reduce mortality rates [3]. Many studies have shown that PSA concentrations can be influenced by a variety of other factors in addition to PCa [4–9]. The aforementioned influencing factors can often lead to overdiagnosis, unnecessary prostate biopsy during screening, and complications of treatment for the indolent disease [3, 10, 11]. Recently, the United States Preventive Services Task Force (USPSTF) updated its recommendation statement from Level D (recommended for against PSA-based prostate cancer screening) to Level C (advocated for a personalized screening approach) [3, 10, 12]. Based on the current evidence, a guideline panel made a weak recommendation against PSA screening for PCa [13]. Since many factors can affect PSA levels, screening for PCa using PSA concentrations remains highly controversial.

A large number of studies strongly indicate that environmental factors play a key role in the pathogenesis of PCa. It is speculated that the prevalence of PCa in Western countries is largely due to the fundamental dietary characteristics of the Western diet patterns [14, 15], which are characterized by high intake of protein, fat, and refined carbohydrates. The World Health Organization (WHO) recommends that countries reduce the burden of noncommunicable diseases by restricting the consumption of “free sugars” [16]. Tumor cells can consume excess glucose via the overexpression of sugar-binding and transporting receptors (mainly GLUT), which allows for the consumption of more energy and greater proliferation than normal cells [17]. Consumption of sugars may be related to PSA concentrations through the activation of inflammatory cytokines as a result of elevated uric acid in the serum (or though other mechanisms) [18]. Since the effect of the total dietary intake of sugars on PSA concentrations is complicated and undefined, there is a compelling need to further evaluate this topic. Hence, we performed a secondary data analysis based on existing data from publicly available data from the US National Health and Nutrition Examination Survey (NHANES). We aimed to explore the relationship between the total dietary intake of sugars and the PSA concentrations.

## 2. Material and Methods

### 2.1. Data Availability

Since 1960, the National Centers for Disease Control (CDC) and Prevention National Health Statistics Center has conducted the NHANES survey every two years to provide estimates of the health and nutritional status of noninstitutional populations in the United States. This data is available on the NHANES website (http://www.cdc.gov/nchs/nhanes/nhanes_questionnairees.htm). The NHANES protocol has been reviewed and approved by the National Center for Health Statistics research ethics review board. All participants provided written informed consent. More detailed information about the NHANES can be found on the official website.

### 2.2. Study Population

**Figure 1:**
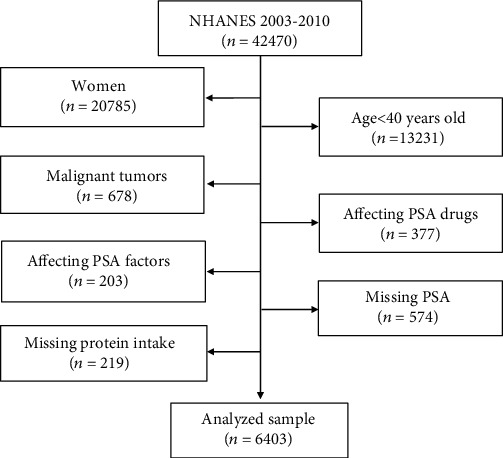
Screening flow chart for male participants (≥40 years) in the US National Health and Nutrition Examination Survey (2003-2010).

In this study, we integrated data from four two-year NHANES survey cycles (2003-2004, 2005-2006, 2007-2008, and 2009-2010) containing PSA data from 2003-2010 and conducted a second analysis. We screened participants according to the following exclusion criteria: (1) female participant (*n* = 20785); (2) aged <40 years (*n* = 13231) [19]; (3) participant with malignant tumors (including PCa patients) (*n* = 678); (4) affecting PSA drugs: using 5ARI or other forms of hormone therapy (i.e., replacing testosterone or castration) and drugs (*n* = 377), with incomplete clinical or sociodemographic data; (5) affecting PSA factors: male with prostatitis or recent prostate surgery (i.e., prostate biopsy and rectal examination within 1 week and surgery or cystoscopy within 1 month) (*n* = 203); and (6) missing PSA and protein intake data (*n* = 793). After concluding the screening, 6,403 out of 42,470 participants were utilized in this study (Figure 1). Besides, this study is the research plan was designed according to the Helsinki Declaration of the World Medical Association (for more information, see the NHANES official website), and since this study is not a clinical trial, it does not require registration.

### 2.3. Variables

In the current study, the target independent variable was total dietary intake of sugars (gm), which was available in the NHANES survey. The US Department of Agriculture (USDA) Automatic Multiple Pass Method (AMPM) was used to collect dietary intake data by interviewers 24 hours a day. A detailed description of the dietary interview method has been described elsewhere [20, 21].

The dependent variable was PSA (ng/mL). Serum samples from participants collected by NHANES physicians were recorded on a Beckman Access using the Hybritech PSA method to record serum total PSA concentrations (ng/mL) (https://wwwn.cdc.gov/Nchs/Nhanes/2001-2002/L11P_2_B.htm). Continuous total PSA data is used as a result variable in our analysis.

Covariates were selected based on previous studies demonstrating the link between these covariates and dietary sugar intake and/or prostate cancer/PSA [4–9, 22–24]. Covariates included demographic, dietary, biological, and immunological variables. Variables included in the database file were as follows: continuous variables included LDL-cholesterol (mg/dL), poverty income ratio (PIR), BMI (kg/m^2^), drinking alcohol first day (gm), vitamin D (ng/mL), C-reactive protein (mg/dL), glycohemoglobin (%), HDL-cholesterol (mg/dL), age (year), and triglycerides (mg/dL). Categorical variables consisted of race, smoked at least 100 cigarettes in life, hypertension history, diabetes history, coronary heart disease, stroke, education level, marital status, physical activity, and enlarged prostate. In general, covariates relate to dietary, comorbidity, physical examination, and demographic data in the NHANES database. A more detailed explanation of these variables can be found on the NHANES official website.

### 2.4. Statistical Analysis

We conducted a statistical analysis according to the criteria of the CDC guidelines (https://wwwn.cdc.gov/nchs/nhanes/tutorials/default.aspx). Total dietary intake of sugars was normally distributed, expressed as the mean ± standard deviation, and continuous variables for data analysis were used. log2 transformation was performed and used as the covariate for data analysis because PSA is a skewed distribution. Continuous variables are expressed as the mean ± standard deviation (normal distribution) or median (quartile) (skew distribution), and categorical variables are expressed in percentage or frequency. To investigate whether the total dietary intake of sugars of a particular participant is related to PSA concentrations, they were first separated into four groups based on the quartile concentrations, and then a weighted chi-square test (categorical variable) or a weighted linear regression model (continuous variable) was used to calculate the difference between the quartile arrays. Secondly, weighted univariate and multiple linear regression models were employed to determine the linear relationship between total dietary intake of sugars and PSA concentrations. Four statistical models were constructed: model I, no adjustment covariates; model II, adjusted only based on social demographic data; and model III, model II + other covariates shown in Table 1. Due to the limitations of linear regression in nonlinearity addressing, a weighted generalized additive model (GAM, Model IV) was also employed to control confounding factors (continuous variables in covariates as nonlinear input equations). Finally, a GAM model and a smooth curve fit (penalty spline method) were performed to explore the nonlinear association between total dietary intake of sugars and PSA concentrations.

To improve the accuracy of the data, the NHANES database was curated to account for missing data (Supplemental table 1). To avoid introducing bias by only including those with complete data, multiple imputations were adopted, which also helps to maximize statistical power [25]. We created five imputed datasets with chain equations using the MICE package [26]. Moreover, we use sensitivity analysis to determine whether the created complete data is significantly different from the preimputation data (Supplemental table 2). Our research indicated that the complete data has no significant difference from the original data. Therefore, all of our multivariate analysis results are based on a calculated dataset and are combined with Rubin's rules.

All analyses were performed using the statistical software package R (http://www.R-project.org, The R Foundation) and EmpowerStats (http://www.empowerstats.com, X&Y Solutions, Inc., Boston, MA). A *P* value of less than 0.05 (two-tailed) was considered to be statistically significant.

## 3. Results

### 3.1. Baseline Characteristics of Participants

Table 2 shows the weighted distribution of demographic sociological characteristics and other covariates selected by NHANES from 2003 to 2010 for selective participants. The average age of participants included in this study was 58.1 years (±13.6). In different total dietary intake of sugar groups (quartiles, Q1–Q4), the distribution of triglycerides, C-reactive protein, BMI, stroke, smoked at least 100 cigarettes in life, and enlarged prostate was approximately similar (all *P* values > 0.05). Compared with the Q4 group, participants with lower intake were older; have lower poverty to income ratio, lower vitamin D, lower LDL-C, and lower physical activity; and are more likely to be lower education level. In contrast, participants in other groups (Q1–Q3) have higher PSA concentrations (log2 transformed), glycohemoglobin, drinking alcohol first day, and single rate and reported a higher incidence of hypertension, diabetes, and coronary heart disease. Most of the participants were non-Hispanic whites.

### 3.2. PSA Concentrations and Total Dietary Intake of Sugars

Table 1 shows the results of the univariate and multiple linear regression models. The nonadjusted model indicated that for each additional unit of total sugars of dietary, the PSA concentrations are reduced by 0.001 (0.001, 0.002) with *P* for trend less than 0.05. In the minimally adjusted model (adjusted for age (year), poverty income ratio, race/ethnicity, education level, and marital status), the association between total sugars of dietary and PSA concentrations was not significant with *P* for trend > 0.05. In the fully adjusted model, after adjusted for age (year), poverty income ratio, race/ethnicity, education level, marital status, VITD, LDL-C, HDL-C, triglycerides, C-reactive protein, glycohemoglobin (%), BMI (Kg/m2), physical activity (MET-based rank) (%), smoked at least 100 cigarettes in life, drinking alcohol (gm) first day, hypertension history, diabetes history, coronary heart disease, stroke, and enlarged prostate, we found that for each additional unit of total dietary intake of sugars, the PSA concentrations are increased by 0.003 (0.001, 0.005) (log2 transformed).

### 3.3. Results of Sensitivity Analysis

**Figure 2:**
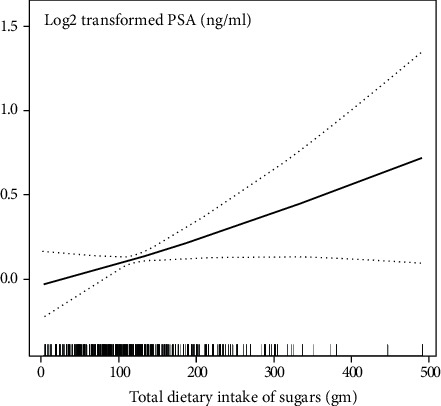
The relationship between total dietary intake of sugars and prostate-specific antigen (PSA) concentrations.

To confirm the stability of the results, a series of sensitivity analyses can performed. Firstly, the total dietary intake of sugars was converted from the continuous variable to the categorical variable in the quartile value and its calculated *P* for trend, which was found to be consistent with the result of the total dietary intake of sugars as a continuous variable. Furthermore, because the generalized linear model is not capable of addressing nonlinearity, the GAM sensitivity analysis was used. The fully adjusted model was observed to be consistent with the GAM model. A smooth curve fit model was used to investigate the possibility of a nonlinear relationship between total dietary intake of sugars and PSA concentrations. After adjusting for other covariates (adjustment strategy with the fully adjusted model), we observed that the relationship between total dietary intake of sugars and PSA concentrations is linear (Figure 2).

We found that for one gram of total dietary intake of sugars, the PSA concentrations are increased by 0.003 ng/mL (log2 transformed) (95% CI: 0.001, 0.005) with *P* for trend less than 0.05. Sensitivity analyses by the GAM model supported this linear association between total dietary intake of sugars and PSA concentrations.

## 4. Discussion

To the best of our knowledge, this is the first study to explore the relationship between total dietary intake of sugars and serum PSA concentrations through a large sample of data in middle-aged men without a history of malignant tumors in the United States. Through the analysis of 6403 NHANES participants, we found that an increase of the total dietary intake of sugars by one gram is associated with increased PSA concentrations of 0.003 ng/mL (log2 transformed) (95% CI: 0.001, 0.005) with *P* for trend less than 0.05. This result was confirmed using a sensitivity analysis and is robust.

The previous survey indicates the added sugars consume an average of 270 calories a day accounting for more than 13% of American energy intake [27]. This intake exceeds the recommendations listed in the 2015-2020 American Dietary Guidelines [27], which emphasizes limiting the intake of added sugars to less than 10% of total caloric intake. Sugars are naturally occurring sweeteners. The most common sugars in the human diet are glucose, fructose, and galactose. Consumption of sugars of dietary intake may be related to PSA concentrations through the activation of inflammatory cytokines, such as interleukins, C-reactive protein, and tumor necrosis factor, among others, as a result of elevated uric acid in the serum or another mechanism [18]. Increased uric acid may in particular lead to increased production of IL-1*β* and chronic inflammation [28]. While naturally occurring sugars and added sugars have the same chemical structure, the difference to be noted may lie in the broader range of effects physiological that ultimately regulate the inflammatory process. It is important to emphasize that processed sugars may have different effects on blood glucose due to the buffering effect of vitamins, fiber, and phytonutrients. The absorption of sugars in plant foods occurs slowly and is more regulated, so blood sugar does not rise suddenly, resulting in the increased inflammatory response [29]. Physiological events associated with the digestion and metabolism of these sugars may in particular lead to an increase or change in proinflammatory cytokines and ultimately to chronic inflammation; this may be the cause of the increased PSA concentrations.

Dietary intake of sugars is associated with a metabolic syndrome (MetS) characterized by elevated blood pressure, triglycerides, LDL cholesterol, uric acid, and inflammation [30–33]. Mets are widespread worldwide and have become a major social and public health issue in the past 20 years. Previous studies have shown that MetS may play a role in serum PSA concentrations [34, 35]. However, the detailed impact of MetS on PSA concentration is unknown, and obesity (waist circumference ≥ 90 cm) is one of the diagnostic indicators of MetS. Previous studies have shown that increased intake of sugar-sweetened beverages is positively correlated with weight gain [36, 37], and other evidence suggests that PSA concentrations are lower in men with higher BMI [7, 38]. However, there is little research evidence suggests that lifestyles (dietary factors) associated with obesity, including energy, fat, protein, and carbohydrate intake affect PSA concentrations. A previous large clinical trial assessed the association between carbohydrate intake and PSA concentrations, but no correlation was found [5]. Ohwaki et al. [39] showed that PSA concentrations were elevated in men with lower protein intake and higher fat intake, but PSA was not associated with total energy or percent carbohydrate intake. However, Parekh et al. [40] reported data from a large number of American males, who did not undergo a recent digital rectal examination or prostate biopsy, had a history of prostate cancer or prostatitis, indicating that higher energy intake was inversely associated with PSA concentrations. But in that study, they did not observe a link between PSA concentrations and the percentage of total energy by fat or carbohydrates, such as total dietary intake of sugars.

Our study indicates that adjusting for dietary, comorbidity, physical examination, and demographic data, the total dietary intake of sugars is positively associated with PSA. To make better use of PSA as a diagnostic tool, we must better understand the nonpathological causes of individual differences in PSA concentrations, and the total dietary intake of sugars is one part that requires further attention.

Compared with the previously published research, this study had some advantages. Firstly, this utilized a large sample volume with a total of 6,403 participants. Secondly, we not only address different types of missing data but also consider the impact of missing data on the results. Thirdly, a sensitivity analysis was performed on the missing data and the scale of impact was evaluated. Fourthly, we use the generalized additive model (GAM) to verify whether the linear relationship was accurate.

Nevertheless, this study does have several limitations that must be considered when interpreting the results. Firstly, due to the intrinsic limitations of the cross-sectional study, it is difficult to distinguish causality. Therefore, further prospective cohort studies are needed to validate the causality. Secondly, we excluded statins, thiazides, NSAIDs, androgen drug [19] users, and malignant tumors participants, because these special populations have a major impact on PSA concentrations. Therefore, the results contained within this study do not apply to the aforementioned populations. Thirdly, the research population is limited to Americans; thus, the generalizability is geographically restricted. Fourthly, this study is based on the secondary analysis of published data so variables that are not included in the data set cannot be adjusted for, such as dihydrotestosterone concentrations. Fifthly, the NHANES database is a cross-sectional study based upon a questionnaire. There is the likelihood of a degree of inherent error in the quantitative estimation of the sugar intake, and this error is difficult to avoid and quantify. Finally, it is regrettable that the specific intake of different sugars is not accounted for within the NHANES database, which makes it impossible to evaluate the distribution of specific sugars and assess which sugars cause the PSA concentrations to change more significantly. This point requires further evaluation in future research.

## 5. Conclusion

After adjusting for dietary, comorbidity, physical examination, and demographic data, the total dietary intake of sugars is independently and positively associated with serum PSA concentrations in adult American males without a history of malignant tumors. However, the overall effects of the total dietary intake of sugars on PCa screening remain unclear and deserve further evaluation. Moreover, it is not yet clear that the total dietary intake of sugars is further involved in the occurrence and development of PCa. It is a difficult question, which also needs further study research for evaluation.

## Figures and Tables

**Table 1:** Univariate and multivariate analyses by the weighted linear regression model and GAM model.

Exposure	Nonadjusted model	Minimally adjusted model	Fully adjusted model	GAM model
Total sugar intake	-0.001 (-0.002, -0.001), <0.00001	-0.000 (-0.000, 0.000), 0.95366	0.003 (0.001, 0.005), 0.00299	0.003 (0.001, 0.005), 0.00291
Total sugar intake				
Q1	ref	ref	ref	ref
Q2	0.047 (-0.057, 0.152), 0.37306	0.073 (-0.029, 0.174), 0.16025	0.260 (-0.422, 0.941), 0.45757	0.260 (-0.422, 0.941), 0.45757
Q3	-0.063 (-0.168, 0.041), 0.23416	0.026 (-0.076, 0.128), 0.62046	0.309 (-0.322, 0.939), 0.34040	0.309 (-0.322, 0.939), 0.34040
Q4	-0.238 (-0.342, -0.134), <0.00001	0.011 (-0.092, 0.114), 0.83292	0.682 (0.122, 1.242), 0.01948	0.682 (0.122, 1.242), 0.01948
P for trend	<0.00001	0.94006	0.01378	0.01337

Nonadjusted model adjust for none. Minimally adjusted model adjust for age (year), poverty income ratio, race/ethnicity, education level, and marital status. Fully adjusted model adjust for age (year), poverty income ratio, race/ethnicity, education level, marital status, VITD, LDL-C, HDL-C, triglycerides, C-reactive protein, glycohemoglobin (%), BMI (kg/m^2^), physical activity (MET-based rank) (%), smoked at least 100 cigarettes in life, drinking alcohol (gm) first day, hypertension history, diabetes history, coronary heart disease, stroke, and enlarged prostate. GAM model adjust for age (year), poverty income ratio, race/ethnicity, education level, marital status, VITD, LDL-C, HDL-C, triglycerides, C-reactive protein, glycohemoglobin (%), BMI (kg/m^2^), physical activity (MET-based rank) (%), smoked at least 100 cigarettes in life, drinking alcohol (gm) first day, hypertension history, diabetes history, coronary heart disease, stroke, and enlarged prostate.

**Table 2:** Baseline characteristics of selected participants.

Total sugar intake (gm)	Q1 (0.14-63.68)	Q2 (63.69-103.86)	Q3 (103.91-156.45)	Q4 (156.64-1061.96)	*P* value
N	1336	1335	1335	1336	
PSA log2 transform (ng/mL)	0.00 (-3.84-5.32)	0.07 (-3.84-5.23)	-0.04 (-3.84-5.32)	-0.20 (-3.84-5.32)	<0.001
Sociodemographic variables					
Age, mean ± SD (years)	61.15 (12.29)	61.03 (12.56)	59.36 (12.74)	54.82 (11.65)	<0.001
Poverty to income ratio, mean ± SD (years)	2.55 (1.58)	2.75 (1.64)	2.85 (1.60)	2.85 (1.63)	<0.001
Race/ethnicity (%)					<0.0001
Mexican American	7.18	6.10	7.79	6.10	
Other Hispanic	4.87	4.13	2.40	2.99	
Non-Hispanic White	70.66	74.78	77.60	79.50	
Non-Hispanic Black	9.73	8.87	9.06	10.18	
Other race/ethnicity	7.56	6.12	3.15	1.23	
Education (%)					<0.0001
Less than high school	10.19	9.14	6.68	4.31	
High school	37.03	30.33	34.70	39.32	
More than high school	52.78	60.53	58.62	56.37	
Marital status (%)					<0.0001
Married	74.18	73.11	73.08	72.68	
Single	21.70	21.05	25.31	19.46	
Living with a partner	4.12	5.84	1.61	7.86	
Variables of laboratory data					
VITD, mean ± SD (ng/mL)	57.83 (22.06)	60.38 (22.39)	61.68 (21.51)	61.88 (21.25)	<0.001
LDL-C, mean ± SD (mg/dL)	113.91 (35.87)	120.84 (37.13)	118.25 (34.96)	119.68 (33.06)	0.004
HDL-C, mean ± SD (mg/dL)	50.03 (16.35)	48.81 (14.33)	48.70 (13.71)	46.66 (13.61)	<0.001
Triglycerides, mean ± SD (mg/dL)	131.50 (32.00-2566.00)	134.00 (21.00-2100.00)	132.00 (24.00-2693.00)	141.00 (26.00-2397.00)	0.054
C-reactive protein, mean ± SD (mg/dL)	0.20 (0.01-18.10)	0.19 (0.01-13.70)	0.19 (0.01-13.90)	0.19 (0.01-18.50)	0.972
Glycohemoglobin (%)	6.16 (1.33)	5.94 (1.21)	5.77 (1.04)	5.72 (1.01)	<0.001
Medical examination and personal life history					
Body mass index, mean ± SD (kg/m^2^)	28.99 (5.63)	28.67 (5.37)	28.82 (5.63)	28.88 (6.14)	0.534
Physical activity (MET-based rank) (%)					<0.0001
Sits	29.08	21.05	18.44	16.24	
Walks	45.04	55.43	46.46	47.66	
Light loads	17.47	17.00	25.16	23.12	
Heavy work	8.41	6.52	9.94	12.98	
Smoked at least 100 cigarettes in life					0.0896
Yes	61.00	57.05	57.12	57.47	
No	39.00	42.95	42.88	42.53	
Dietary interview—individual foods					
Drinking alcohol (gm) first day	19.29 (39.28)	14.37 (34.47)	12.57 (28.96)	10.93 (30.12)	<0.001
Comorbidities (%)					
Hypertension history					<0.0001
Yes	46.56	46.54	34.17	36.03	
No	53.44	53.46	65.83	63.97	
Diabetes history					<0.0001
Yes	24.52	14.01	7.93	3.17	
No	75.48	85.99	92.07	96.83	
Coronary heart disease					0.0314
Yes	8.75	9.42	5.22	5.80	
No	91.25	90.58	94.78	94.20	
Stroke					0.5012
Yes	3.82	4.21	2.96	2.60	
No	96.18	95.79	97.04	97.40	
Enlarged prostate					0.0753
Yes	14.92	20.38	15.97	14.60	
No	85.08	79.62	84.03	85.40	

Q1–Q4: grouped by quartile according to the total sugar content of the dietary intake group.

## Data Availability

Data can be downloaded from the “NHANES” database (https://www.cdc.gov/nchs/nhanes/index.htm).

## Abbreviations

NHANES: National Health and Nutrition Examination Survey
PSA: Prostate-specific antigen
PCa: Prostate cancer
GAM: Generalized additive model
CI: Confidence interval.

## Supplementary Materials

Supplementary 1 Additional file 1: Supplemental table 1: the description of missing data.

Supplementary 2 Supplemental table 2: sensitivity comparative analysis between preimputation and postimputation.
