# Supplementary material for: Association of Total Dietary Intake of Sugars with Prostate-Specific Antigen (PSA) Concentrations: Evidence from the National Health and Nutrition Examination Survey (NHANES), 2003-2010
Source: Biomed Res Int. 2021 Jan 9;2021:4140767. doi: 10.1155/2021/4140767 (PMC7811566; doi:10.1155/2021/4140767)
Supplement: Supplementary 1 — Additional file 1: Supplemental table 1: the description of missing data. [file 4140767.f1.pdf]

Supplemental table 1: Covariate missing scale.

Variables	Non-missing	Missing
Age,year	6622	0
Poverty income ratio (%)	6187	435
Race/Ethnicity (%)	6622	0
VITD（ng/mL）	6437	185
LDL-C (mg/dL)	3053	3569
HDL (mg/dL)	6622	0
Triglycerides (mg/dL)	6603	19
C-reactive protein(mg/dL)	6621	1
Body mass index,Kg/m^2	6509	113
Smoked at least 100 cigarettes in life	3854	2768
Drinking alcohol (gm) first day	6344	278
Hypertension history	3939	2683
Diabetes history	3838	2784
Coronary heart disease	3864	2758
Stroke	3864	2758
Enlarged prostate	4788	1834
Lead (umol/L)	6617	5
Cadmium (nmol/L)	6617	5
Mercury, total (umol/L)	6617	5
