# Supplementary material for: Association of Total Dietary Intake of Sugars with Prostate-Specific Antigen (PSA) Concentrations: Evidence from the National Health and Nutrition Examination Survey (NHANES), 2003-2010
Source: Biomed Res Int. 2021 Jan 9;2021:4140767. doi: 10.1155/2021/4140767 (PMC7811566; doi:10.1155/2021/4140767)
Supplement: Supplementary 2 — Supplemental table 2: sensitivity comparative analysis between preimputation and postimputation. [file 4140767.f2.pdf]

Supplemental table 2: Sensitivity comparative analysis between pre-imputation and post-imputation

MI.ITER	pre-imputation	1st post-imputation	2nd post-imputation	3rd post-imputation	4th post-imputation	5th post-imputation	P-value	P-value*
Sociodemographic variables								
Age, mean ± SD (years)	59.47 (12.79)	59.47 (12.79)	59.47 (12.79)	59.47 (12.79)	59.47 (12.79)	59.47 (12.79)	1.000	1.000
Poverty to income ratio, mean ± SD (years)	2.74 (1.61)	2.72 (1.61)	2.73 (1.62)	2.73 (1.63)	2.72 (1.63)	2.73 (1.62)	0.976	0.995
Race/Ethnicity(%)							1.000	-
Mexican American	1239 (18.71%)	1239 (18.71%)	1239 (18.71%)	1239 (18.71%)	1239 (18.71%)	1239 (18.71%)		
Other Hispanic	387 (5.84%)	387 (5.84%)	387 (5.84%)	387 (5.84%)	387 (5.84%)	387 (5.84%)		
Non-Hispanic White	3504 (52.91%)	3504 (52.91%)	3504 (52.91%)	3504 (52.91%)	3504 (52.91%)	3504 (52.91%)		
Non-Hispanic Black	1241 (18.74%)	1241 (18.74%)	1241 (18.74%)	1241 (18.74%)	1241 (18.74%)	1241 (18.74%)		
Other race/ethnicity	251 (3.79%)	251 (3.79%)	251 (3.79%)	251 (3.79%)	251 (3.79%)	251 (3.79%)		
Education (%)							1.000	-
Less than high schoo	1155 (17.47%)	1157 (17.47%)	1158 (17.49%)	1159 (17.50%)	1160 (17.52%)	1157 (17.47%)		
High school	2488 (37.63%)	2493 (37.65%)	2493 (37.65%)	2491 (37.62%)	2490 (37.60%)	2494 (37.66%)		
More than high school	2969 (44.90%)	2972 (44.88%)	2971 (44.87%)	2972 (44.88%)	2972 (44.88%)	2971 (44.87%)		
Marital Status (%)							1.000	-
Married	4493 (67.94%)	4500 (67.96%)	4500 (67.96%)	4498 (67.93%)	4500 (67.96%)	4500 (67.96%)		
Single	1788 (27.04%)	1790 (27.03%)	1790 (27.03%)	1791 (27.05%)	1790 (27.03%)	1790 (27.03%)		
Living with partner	332 (5.02%)	332 (5.01%)	332 (5.01%)	333 (5.03%)	332 (5.01%)	332 (5.01%)		
Variables of laboratory data								
VITD（ng/mL）	60.21 (21.72)	60.23 (21.79)	60.27 (21.69)	60.28 (21.66)	60.29 (21.70)	60.28 (21.71)	1.000	1.000

LDL-C (mg/dL)	118.60 (35.22)	119.78 (35.67)	119.46 (35.67)	118.81 (35.57)	119.16 (35.90)	119.32 (35.59)	0.581	0.270
HDL-C (mg/dL)	48.58 (14.37)	48.58 (14.37)	48.58 (14.37)	48.58 (14.37)	48.58 (14.37)	48.58 (14.37)	1.000	1.000
Triglycerides (mg/dL)	133.00 (21.00-2693.00)	133.00 (-295.03-2693.00)	133.00 (-75.64-2693.00)	133.00 (-175.92-2693.00)	133.00 (-208.16-2693.00)	133.00 (-93.90-2693.00)	1.000	1.000
C-reactive protein (mg / dL)	0.19 (0.01-18.50)	0.19 (0.01-18.50)	0.19 (0.01-18.50)	0.19 (0.01-18.50)	0.19 (0.01-18.50)	0.19 (0.01-18.50)	1.000	1.000
Glycohemoglobin (%)	5.89 (1.17)	5.89 (1.17)	5.89 (1.17)	5.89 (1.17)	5.89 (1.17)	5.89 (1.17)	1.000	1.000
Medical examination and personal life history								
Body mass index (Kg/m^2)	28.75 (5.55)	28.77 (5.55)	28.76 (5.55)	28.76 (5.55)	28.75 (5.56)	28.76 (5.55)	1.000	1.000
Physical Activity (MET-based rank) (%)							0.077	-
Sits	991 (26.19%)	1508 (22.77%)	1548 (23.38%)	1607 (24.27%)	1588 (23.98%)	1629 (24.60%)		
Walks	1867 (49.34%)	3227 (48.73%)	3183 (48.07%)	3195 (48.25%)	3243 (48.97%)	3239 (48.91%)		
Light loads	617 (16.31%)	1253 (18.92%)	1263 (19.07%)	1220 (18.42%)	1189 (17.96%)	1161 (17.53%)		
Heavy work	309 (8.17%)	634 (9.57%)	628 (9.48%)	600 (9.06%)	602 (9.09%)	593 (8.95%)		
Smoked at least 100 cigarettes in life							0.609	-
Yes	2431 (63.08%)	4108 (62.04%)	4104 (61.98%)	4055 (61.24%)	4092 (61.79%)	4100 (61.91%)		
No	1423 (36.92%)	2514 (37.96%)	2518 (38.02%)	2567 (38.76%)	2530 (38.21%)	2522 (38.09%)		
Dietary interview - individual foods								
Drinking alcohol (gm) first day	14.40 (34.34)	14.31 (34.45)	14.23 (34.37)	14.48 (34.49)	14.42 (34.28)	14.43 (34.27)	0.999	0.968
Comorbidities (%)								
Hypertension history							0.221	-
Yes	1671 (42.42%)	3076 (46.45%)	3080 (46.51%)	3064 (46.27%)	3198 (48.29%)	3202 (48.35%)		

No	2268 (57.58%)	3546 (53.55%)	3542 (53.49%)	3558 (53.73%)	3424 (51.71%)	3420 (51.65%)		
Diabetes history							0.191	-
Yes	626 (16.31%)	1351 (20.40%)	1298 (19.60%)	1319 (19.92%)	1318 (19.90%)	1387 (20.95%)		
No	3212 (83.69%)	5271 (79.60%)	5324 (80.40%)	5303 (80.08%)	5304 (80.10%)	5235 (79.05%)		
Coronary heart disease							0.063	-
Yes	283 (7.32%)	833 (12.58%)	795 (12.01%)	795 (12.01%)	803 (12.13%)	770 (11.63%)		
No	3581 (92.68%)	5789 (87.42%)	5827 (87.99%)	5827 (87.99%)	5819 (87.87%)	5852 (88.37%)		
Stroke							0.082	-
Yes	180 (4.66%)	425 (6.42%)	482 (7.28%)	482 (7.28%)	469 (7.08%)	515 (7.78%)		
No	3684 (95.34%)	6197 (93.58%)	6140 (92.72%)	6140 (92.72%)	6153 (92.92%)	6107 (92.22%)		
Enlarged prostate							0.809	-
Yes	836 (17.55%)	1180 (17.82%)	1142 (17.25%)	1125 (16.99%)	1127 (17.02%)	1145 (17.29%)		
No	3928 (82.45%)	5442 (82.18%)	5480 (82.75%)	5497 (83.01%)	5495 (82.98%)	5477 (82.71%)		

Note: This result indicates that there is no difference in the distribution of data after interpolation before interpolation, that is, interpolation does not cause real data changes.
